# Recent Progress on Creep Properties of ODS FeCrAl Alloys for Advanced Reactors

**DOI:** 10.3390/ma16093497

**Published:** 2023-05-01

**Authors:** Haodong Jia, Yingjie Wang, You Wang, Lu Han, Yujuan Zhang, Zhangjian Zhou

**Affiliations:** School of Materials Science and Engineering, University of Science and Technology Beijing, Beijing 100083, China

**Keywords:** ODS FeCrAl alloy, accident tolerant fuel, creep properties

## Abstract

In order to meet the growing energy demand, more environmentally friendly and efficient GEN-IV reactors have emerged. Additionally, nuclear structural materials need larger more safety margins for accident scenarios as a result of the Fukushima accident. In order to extend the failure time and lessen the effect of accidents, this design method for accident-tolerant fuel materials calls for cladding materials to maintain good corrosion resistance and mechanical properties during a beyond design basis accident (BDBA). Accidents involving nuclear reactors would undoubtedly result in higher temperatures, which would make it much harder for materials to withstand corrosion. Oxide dispersion strengthened (ODS) FeCrAl alloys have shown promise as candidate materials because of their extraordinarily slow reaction rates under high-temperature steam. However, the addition of the Al element renders the alloy’s high-temperature mechanical properties insufficient. In particular, studies on the alloy’s creep properties are extremely rare, despite the fact that the creep properties are crucial in the real service environment. Therefore, this paper focuses on the creep properties of ODS FeCrAl alloy, summarizes and analyzes the research results of this material, and provides a reference for future research and applications.

## 1. Introduction

Global energy needs are rising along with the world’s ongoing population growth. Energy supply assistance is necessary to raise people’s living standards, health levels, and life expectancies. Nuclear energy is a clean, dependable, and efficient solution to the world’s energy supply issues, as well as environmental issues. The Gen-II reactors currently in operation are nearing the end of their lifespan, and it will take some time before the Gen-III reactors currently being built can provide the anticipated levels of electricity. Therefore, a new generation of cleaner, more effective, and safer reactors is required at this pivotal time. The design, known as a Gen-IV nuclear energy system, was presented at the International Forum on Gen-IV nuclear reactors. Compared with previous generations of nuclear energy systems, the Gen-IV advanced nuclear energy systems have improved in terms of economy, efficiency, environmental friendliness, and safety. However, the corresponding working conditions have also become more stringent, whether it is service temperature or radiation dose, which has substantially improved. Due to the Fukushima nuclear power plant accident, new ideas are emerged for the design of nuclear structural materials, which should delay fuel degradation and mitigate accident outcomes during a beyond design basis accident (BDBA) Traditional zirconium alloys and stainless steels have been difficult to serve in the fourth generation of advanced nuclear energy systems. Therefore, there is a demand for fuel that is accident-tolerant, yet still has enough power to delay failure and lessen the effects of the mishap.

Oxide dispersion strengthened (ODS) FeCrAl alloy, produced through a powder metallurgical process, has emerged as a very attractive candidate material for advanced energy systems and accident-tolerant fuel (ATF) materials. The ODS iron-based alloy is a crucial substance with growth potential for cladding tube materials. One advantage is that it has great mechanical qualities at high temperatures and radiation resistance due to the numerous dispersed particles. As a material with excellent corrosion resistance, FeCrAl alloy is also widely used in electrothermal alloys that require high temperature and corrosion-resistant environments. Therefore, the ODS material of FeCrAl alloy naturally has great potential as an ATF material.

In the present review, the ODS FeCrAl maintained expectations for the superposition of its excellent properties at the beginning of the design, but after the material was prepared, corresponding problems emerged. The Al element was originally designed to improve corrosion resistance, but after the introduction of the ODS FeCr alloy, it also participated in the formation of dispersed particles, resulting in changes in the original Y–Ti–O dispersed particle system. The size of the dispersed particles of Y–Al–O, in contrast, is larger, reducing the number density of the dispersed particles, which also causes a decline in the high-temperature mechanical properties of the material. Therefore, how to make the ODS FeCrAl alloy have excellent corrosion resistance while maintaining its high-temperature mechanical properties has become a top priority. Furthermore, creep performance that is more suitable for the actual service environment is particularly important. Creep is a crucial consideration for nuclear structural materials since these materials are exposed to high temperatures and stress over an extended period of time, which can lead to creep deformation and failure. However, it is challenging to manufacture ODS FeCrAl alloys in large quantities; there are numerous elements that might affect creep qualities, and the precise creep mechanism is still being researched. As a result, the creep properties of ODS FeCrAl alloys are not thoroughly understood. Therefore, this review summarizes the current research on the creep properties of ODS FeCrAl alloys based on the urgent need for this key material, providing data sorting and support for its practical application.

## 2. Background and Microstructure of ODS FeCrAl Alloy

### 2.1. Development History of ODS FeCrAl Alloy

**Figure 1:**
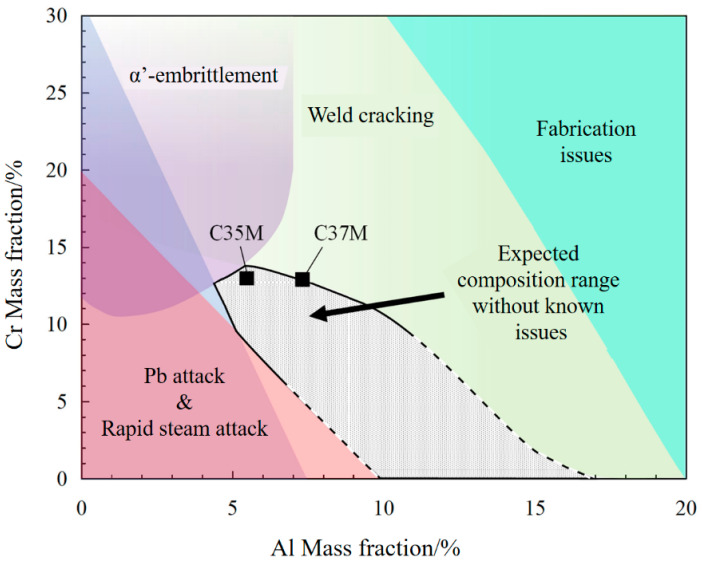
Composition design space for FeCrAl alloys.

The FeCrAl alloy has a significant application value in high-temperature materials due to its strong high-temperature corrosion resistance, which is also evident in its early widespread use as an electrothermal alloy. The third element effect of the Cr and Al in FeCrAl alloys is the source of their superior corrosion resistance compared to other iron-based alloys [1]. Al must be added in order to generate protective Al_2_O_3_ coating because the typical FeCr alloy’s protective Cr_2_O_3_ layer is difficult to sustain above 1100 °C. In order to realize the selective oxidation of Al, a sufficient amount of Cr is the prerequisite [2]. After the Cr-free FeAl alloy is oxidized at 1000 °C for 1 min, a thick oxide film mainly composed of Fe will be formed on the surface [3]. The excellent protection of this oxide layer is more fully reflected in the high temperature Li–Pb. In the study of Pint et al., the extremely low mass loss of 12Cr5Al–ODS alloy in Li–Pb at 700 °C was not achieved by ODS–FeCr alloy [4]. The early FeCrAl alloys were developed from FeCr alloys, and these early FeCr alloys usually contained higher Cr content for better corrosion resistance, generally between 18–30 wt.%. However, too high Cr content will lead to the precipitation of the α’ phase in the alloy at high temperature, resulting in the overall brittleness of the alloy. Another reason for the high temperature brittleness of FeCrAl alloy is that the Al-rich particles are easy to grow [5]. Therefore, there is a concerted effort in current research to reduce the Cr content in FeCrAl alloys. The Cr content in widely commercial APMT alloys was reduced from 30% to 20.5 wt.%, and the Cr content in ALKROTHAL 3 alloys was reduced to 12 wt.%. Finally, through much research, the approximate range of Cr and Al content, under the premise of maintaining the corrosion resistance of the alloy, was obtained. The former is generally between 5–14 wt.%, and the latter is generally between 4–10 wt.%. Combining various properties also proposes a compositional design space, as shown in Figure 1 [6,7,8].

Since discovering that the ODS alloy prepared by the mechanical alloying method has excellent mechanical properties, a variety of ODS alloy systems have been developed. The earliest ODS FeCrAl alloy MA956 was developed and used for more than 40 years [9,10]. Because MA956 still has good corrosion resistance and high strength above 1100 °C, it has been widely used as a superalloy in the structural materials of gas turbine combustors. PM2000, which has a similar composition, is also often used as a heat exchanger [11]. ODS FeCrAl alloy became a research hotspot again after the Fukushima accident in 2011. Prior to this, ODS FeCr alloys were widely studied as structural materials for advanced nuclear energy systems because of their high number density dispersed particles and good radiation resistance. Interest especially grew after it was discovered that adding Ti to the ODS FeCr alloy can form Y–Ti–O particles with only a few nanometers [12,13,14,15]. However, the Fukushima accident drew attention to the performance of structural materials in nuclear energy systems under extreme conditions, and led to the design of accident tolerant fuel [16,17,18]. The high-temperature steam corrosion resistance of structural materials was given high priority due to the loss of coolant accident (LOCA). For this reason, the ODS FeCrAl alloy, which can maintain a very low reaction rate even at a high temperature above 1400 °C, once again became a research topic in structural materials of advanced nuclear energy systems [19]. The major elements of several FeCrAl alloys currently studied are shown in Table 1.

### 2.2. Microstructure of ODS FeCrAl Alloy

Due to the addition of Al, the dispersion system inside the ODS alloy has undergone tremendous changes. In fact, this has been found in the early research on the ODS FeCrAl alloy. The average particle size of the dispersed particles in the MA956 alloy is 22 nm [26]. This is because Al is more likely to react with Y_2_O_3_ and generate Y–Al–O particles compared to Ti [27]. The structure of Y–Al–O particles is more complicated than that of Y–Ti–O, and four kinds of complex dispersed particles were characterized in ODS FeCrAl alloys [28,29,30], namely YAlO_3_ (YAP), Y_3_Al_5_O_12_ (YAG), Y_4_Al_2_O_9_ (YAM) and YAlO_3_ (YAH). Moreover, the particle size of the YAG and YAP structures is larger, usually tens of nanometers. These large-sized Y–Al–O particles lead to an increase in the average size of the dispersed particles inside the alloy and a decrease in the number density [31]. In ODS FeCrAl alloy, Y–Al–O particles are easier to form both from a thermodynamic and kinetic point of view. The excellent high-temperature mechanical properties and radiation resistance of ODS alloys are closely related to their high-number density fine-dispersed particles, so the corresponding properties of early ODS FeCrAl alloys were not outstanding [32,33].

**Figure 2:**
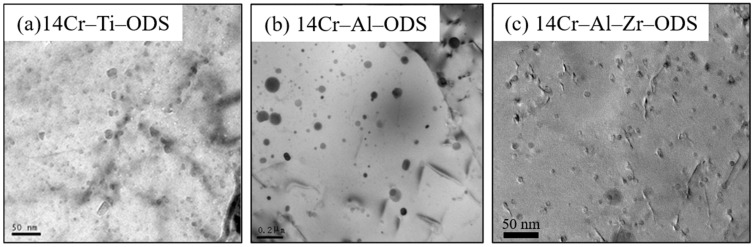
Effects of Al and Zr elements on dispersed particles in ODS alloys. (**a**) 14Cr–Ti–ODS alloy, (**b**) 14Cr–Al–ODS alloy, (**c**) 14Cr–Al–Zr–ODS alloy.

Studies [34,35,36] have shown that the Zr element is more likely to react with Y_2_O_3_ than the Al element. Adding the Zr element to the ODS FeCrAl alloy can change its microstructure: the type of dispersed particles changes from Y–Al–O to Y–Zr–O (mainly Y_4_Zr_3_O_12_), the size of Y–Zr–O particles is significantly finer than Y–Al–O particles, and the corresponding number density also improves [37]. Previous work has also confirmed that the addition of Zr content at 0.6 wt.% had the smallest average particle size and the highest mechanical properties [25]. Moreover, there are studies to change the type of dispersed particles by adding the Hf element to the ODS FeCrAl alloy. The outcomes demonstrated the formation of Y–Hf–O-dispersed particles with lower particle sizes [38]. In addition, because the properties of Y and Zr elements are similar, some researchers proposed using ZrO_2_ to replace the previous dispersion system dominated by complex Y–Al–O particles [39]. The TEM images of ODS alloys containing different dispersed particles are shown in Figure 2, in which 14Cr–Ti does not contain Al (a), the dispersed particles become coarser after adding Al (b), but this trend changes again after adding Zr (c) [40,41].

In addition to optimizing the composition design, the microstructure can also be adjusted by changing the preparation process. A large number of studies have shown that dispersed particles in ODS alloys are mainly formed during the annealing of mechanically alloyed powders at temperatures above 900 °C [42,43]. By changing the addition strategy of Al elements in the ball milling process, Xu [44] effectively improved the uniformity of Al and the ratio of Y/Al to control the type of Y–Al–O particles.

## 3. The Interaction of Dislocations and Dispersed Particles in Creep

Dispersion strengthened alloys have always been known for their high temperature mechanical properties. The large number of dispersed particles is the source of their excellent properties. It is precisely because of the existence of dispersed particles that their creep mechanisms are different. In general, there are generally two mechanisms for the source of particle-strengthened alloys, namely the Friedel cutting mechanism and the Orowan bypassing mechanism. The former generally corresponds to coherent particles, and the latter generally corresponds to incoherent particles. Through the Orowan mechanism, the yield strength of materials at low temperatures can be closely predicted. However, when the temperature rises, the strengthening part calculated by Orowan is often larger than the actual value. This is mainly because dislocations can overcome dispersed particles by climbing at high temperature. Therefore, dispersion strengthening provides a stronger strengthening effect under high temperature and low stress conditions, than under low temperature and high stress conditions.

**Figure 3:**
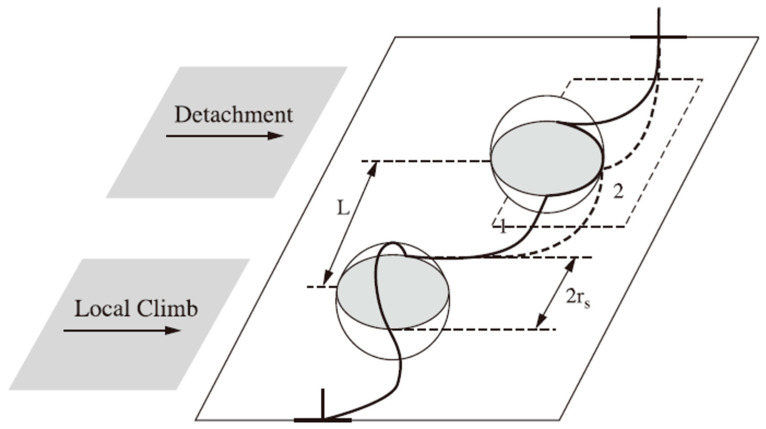
Two mechanisms of interaction between dislocations and dispersed particles.

This special mechanism makes typical ODS alloys have a threshold stress during the creep process. Normally, the sample does not deform plastically when the applied stress is below the threshold stress. There are two theories about the threshold stress: one is the threshold generated by the increase of the total length of the dislocation during the movement process, and the other is the detachment stress of the dislocation after climbing through the dispersed particles. The mechanisms are shown in Figure 3 [45].

Ansell and Weertman [46] believed that dislocation climbing was allowed to occur at higher temperature and lower stress a long time ago. Furthermore, the increase of elastic strain energy associated with the increase of dislocation line length is generally considered to be the key factor leading to the threshold stress. Arzt and Ashby proposed the following formula as the criterion:

$$\tau_{c}=Gb∕L\left(0.5\alpha^{\prime }\right)$$

where α’ is the climb resistance of dislocation climbing, which is related to the volume fraction of dispersed particles and particle diameter, and *τ_c_* is the threshold stress. However, this stress is a very small part of the Orowan stress, almost less than 20%.

In addition to the climbing mechanism, many researchers have observed through in-situ TEM that dislocations need to be separated after climbing on the dispersed particles [47,48]. This may be because the incoherent dispersed particles have some interfaces that can slide and attract dislocations by reducing the total elastic strain energy. Therefore, the creep process reflects that when dislocations leave the interface, it is necessary to overcome the attraction of dispersed particles. On this point, Arzt and coworkers [49,50] analyzed the detachment process and described the stress as:

$$\tau_{d}={\left[1-k_{R}^{2}\right]}^{1∕2}\left(Gb∕L\right)$$

where *k_R_* is the relaxation factor. For iron-based alloys, the value of *k* is generally between 0.93 and 0.98, so it can be seen that the separation stress generated by thermal activation is much greater than the stress required by dislocation climbing.

## 4. Creep of ODS FeCrAl Alloy

### 4.1. Thermal Creep Properties

High-temperature creep performance is an important service performance of nuclear structural materials, but the current research on the creep performance of ODS FeCrAl alloys is still relatively lacking. On the one hand, this is because the characterization of creep performance is relatively complex, and under higher temperature conditions, multiple creep mechanisms may occur simultaneously. Coupled with the large number of dispersed particles in the ODS alloy, the quantification of this process becomes very complicated. In addition, in the characterization of creep performance, a large number of parameters are empirical parameters, which also makes it very difficult to predict creep performance from a non-phenomenological point of view. On the other hand, because ODS alloys are mostly prepared by mechanical alloying and subsequent sintering, such as hot isostatic pressing or hot extrusion, this makes their mass production a problem. Therefore, the current research on the creep properties of ODS FeCrAl alloy is still in the stage of non-standardized process. Different sample preparation processes and different sample heat treatment histories all have a great influence on the creep properties. Not only that, but the size specification of creep samples and the manner of applying stress also make the samples have different performances. For example, some researchers conduct creep experiments through hoop stress, while others perform creep performance characterization through burst experiments. This also makes it difficult to make a direct horizontal comparison of the results of creep properties and there is a large gap between them. However, the relevant research on the creep properties of ODS FeCrAl alloys can still provide enlightenment. It is encouraging that ODS FeCrAl alloy performs very well in terms of creep under high temperature and low stress conditions [51,52].

**Figure 4:**
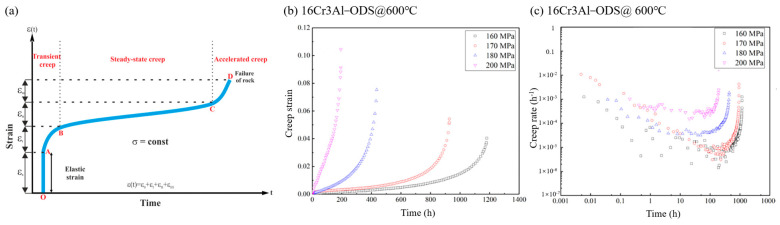
Creep process. (**a**) Schematic diagram of creep process (OA: elastic strain, AB: transient or primary creep, BC: steady state creep, CD: accelerated or tertiary creep), (**b**) creep strain versus time curves of 16Cr3Al–ODS alloys, (**c**) creep rate versus time curves of 16Cr3Al–ODS alloys.

In the creep performance research of many ODS alloys, we can find that the curves of creep strain and creep time do not conform to the typical three-stage creep, as shown in Figure 4 [53,54]. This was found in the study by Lobb et al. [55]. This phenomenon is usually reflected in the fact that there is no clear boundary between the steady-state creep stage and the accelerated creep, or that there is no steady-state creep region. This phenomenon was also observed in the study by Jaumier et al. [56]. Not limited to ODS FeCrAl alloy, this phenomenon was also observed in MA957 alloy [57], and even in ODS-310 alloy [58]. Although this point has been examined in previous research on the creep of ODS alloys, it has not been analyzed in depth. In view of the fact that this phenomenon widely exists in ODS alloys of various compositions, it may be that the creep rate of ODS alloys during the creep process is mainly determined by a large number of dispersed particles inside, causing the samples to directly fracture in the second stage.

ODS alloys are usually prepared by hot extrusion or hot isostatic pressing and subsequent heat treatment. A large number of dispersed particles inside make it difficult to recrystallize, so the anisotropy produced during the preparation process is usually retained. As early as 1978, Whittenberger and coworkers [10] discovered the directionality of the creep properties of the alloy through the creep study of the MA956E alloy. The creep performance is better when the stress loading direction is consistent with the grain aspect ratio long dimension direction, which has also been reported in many studies [59], and the difference in steady creep rate is even hundreds of times different in these two directions [60]. Therefore, the grain aspect ratio is also a very important parameter when studying the creep properties of ODS FeCrAl alloy. In the study of Salmon et al. [61], it was also proposed that this is due to the fact that the elongated grain direction is more likely to transmit cracks, so the gap caused by anisotropy should be examined during the application of ODS alloys.

Klueh’s research found that the creep performance of ODS alloy without Ti is relatively poor, not as good as the two commercial ODS FeCrAl alloys, MA956 and PM2000 [15]. It was later found that this was because the introduction of Ti greatly increased the number density of dispersed particles in the sample, which was also verified in the study of Ohtsuka et al. [62]. They also further studied the relationship between Ti content and creep performance, and finally determined that the Ti content of 0.3–0.35 has the best creep performance, which is one of the reasons why the Ti content of ODS FeCr alloys is generally within this range. The comparison with ODS EUROFER 97 also reflects the importance of Ti content [63]. The N content is also closely related to the creep properties of ODS alloys. In Oka et al.’s [64] research, creep properties are very sensitive to the N content, and changes in a small range will cause large differences in creep properties. This is mainly because the solubility of N in austenite is greater than that of ferrite, so it will be enriched on the boundary of the two phases during rolling and form precipitates with Ti, thereby reducing the refining effect of Ti on dispersed particles, and ultimately affect creep properties. Although there is no effect of N content on the creep properties of ODS FeCrAl alloys, it is well known that N easily reacts with Al and forms precipitates, so the effect of N on the creep of ODS FeCrAl alloys may have different results.

**Figure 5:**
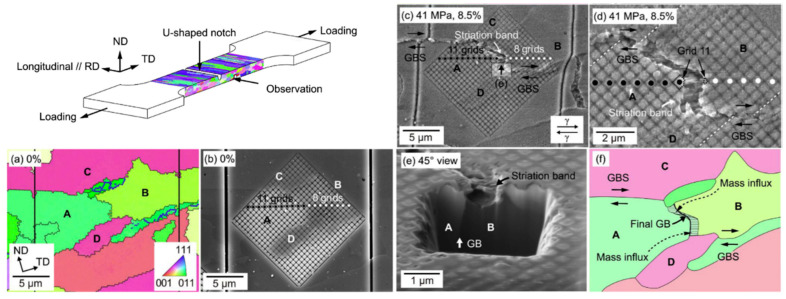
Schematic diagram of the sample and IPF and SEM photos before and after creep at 900 °C and 41 MPa. (**a**) IPF map and (**b**) SEM micrograph before deformation; (**c**,**d**) SEM micrograph at different magnifications after deformation; (**e**) SEM micrograph on an FIB trench between grains A and B, (**f**) schematic diagram of the mass flux.

However, the effect of grain size is not as obvious as that of grain shape, which may be due to the existence of a large number of dispersed particles that do not allow the intragranular diffusion to play a decisive role. In the study of Hayashi [65], it was found that the small-sized grains did not have obvious grain boundary sliding during the creep process. Although the grains grow during prolonged high-temperature creep, the steady-state creep rate does not seem to be very sensitive to the grain size. Moreover, the occurrence of grain boundary sliding is often under high temperature and low stress conditions, which has been confirmed in the research of Masuda [66] who performed creep experiments on 15Cr4Al–ODS alloy at 900 °C and 41 MPa. Meanwhile, grid calibration was carried out by FIB in advance, and EBSD characterization was carried out before and after creep, as shown in Figure 5. The experimental results clearly show that the rate of grain boundary sliding is completely controlled by the mass translation via the grain boundary, and the deformation was mainly caused by the cooperative process of grain boundary sliding and diffusional accommodation, while the deformation in the grain was negligible. It is worth noting that the loading direction in this experiment is parallel to the transverse direction (TD), which is different from the usual parallel to the rolling direction (RD). However, it can still be observed that the direction of mass influx is still along the long axis of the elongated grains, which also shows that the grain shape has a great influence on the creep performance. In addition to the grain size, the influence of the size of the dispersed particles has not been agreed on so far. It is generally believed that the volume fraction and average spacing of dispersed particles have a greater impact. Early studies have shown that the size of dispersed particles has little effect on dislocation climbing [50], and on the steady-state creep rate above 600 °C [67].

**Figure 6:**
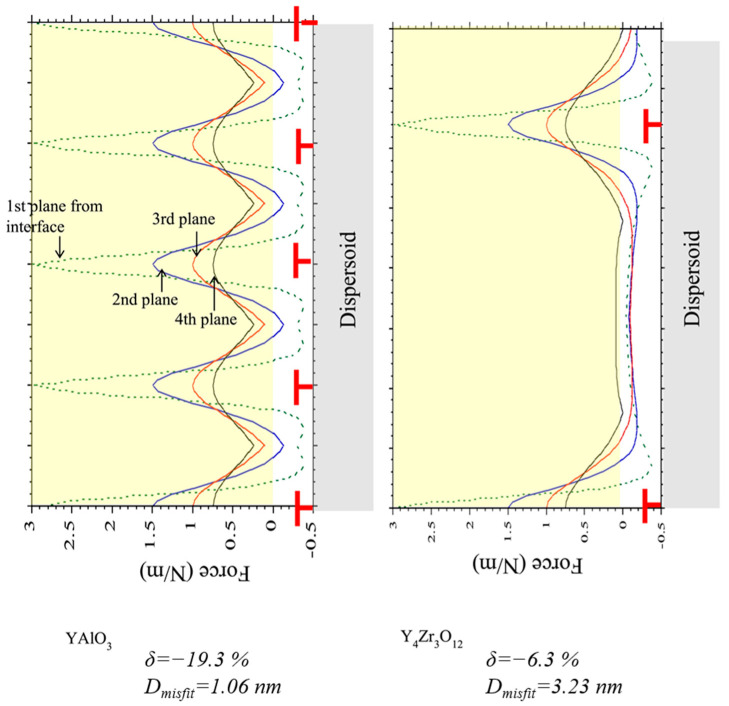
The calculational results of force acting on the dislocation along x-direction for YAlO_3_ and Y_4_Zr_3_O_12_ dispersoids.

In 2020, Ukai et al. [68] studied the creep properties of three ODS alloys containing different dispersed particles, namely Y–Ti–O, Y–Zr–O, and Y–Al–O particles, under ultra-high temperature conditions. And the threshold stress calculation methods suitable for the three kinds ODS alloys were provided respectively. The threshold stress calculated through the detachment mechanism was very close to the actual measured value, but this is still a phenomenological relationship. This study verified that the deformation mechanism was grain boundary sliding under low stress conditions and dislocation climbing under medium stress conditions. In subsequent studies [69], a more in-depth study was carried out on the relationship between the effect of local stress on dislocation movement and the creep rate caused by the mismatch between different dispersed particles and the matrix. This provides a new idea for the characterization of the creep properties of ODS FeCrAl alloys. As shown in Figure 6, the misfit degree of different dispersed particles and the matrix interface is different, so the dislocation spacing will also be different, and different interface relationships have different effects on the dislocations moving over. In this study, it was shown that the smaller coherence (YAlO_3_ particles) would generate normal stress, which in turn would increase the creep rate. However, more research is needed to determine the specific effect of different local stresses on creep rate, which will also become more difficult due to the complex dispersed particle system in ODS FeCrAl alloys.

**Figure 7:**
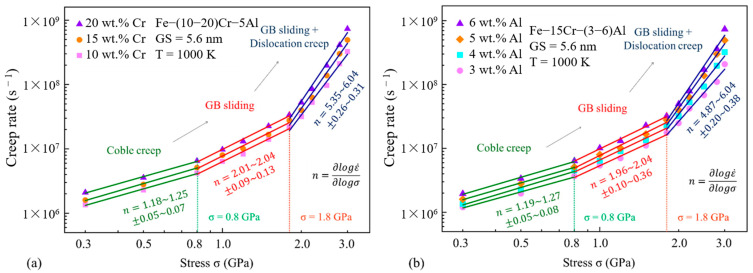
Double logarithm plot of the creep rate vs. stress for FeCrAl samples with (**a**) varying mass fractions of Cr, and (**b**) varying mass fractions of Al.

The stress exponent n serves as another crucial variable in the study of the creep behavior of ODS FeCrAl alloys. Because of the threshold stress, ODS alloys exhibit high stress sensitivity during creep, especially when the applied stress is close to the threshold stress. This also leads to the high stress exponent of ODS alloys. For example, MA957 has approximately 20 [57], 18Cr–ODS has reached 26 [70], and some studies have even obtained a stress index of 125 in 14Cr–ODS [56]. This is very different from traditional metal materials. As previously mentioned, most metal materials follow a 5-power law relationship, and the stress exponent is 5. This rule has also been verified in the non-ODS FeCrAl alloy [55,71,72]. However, the stress exponent of the ODS FeCrAl alloy seems to be different from that of the previous ODS alloy. The stress exponent of the sample in Ren’s research is 4 [54], but it is worth noting that the sample is a forged state. This has also been verified in Ukai’s research, whose ODS alloy containing Y–Al–O dispersed particles has a stress exponent of 6 [68]. The improvement of Zr on the creep properties of ODS FeCrAl alloys has also been verified in Kamikawa’s research [73]. In Yao’s simulation of the creep behavior of FeCrAl alloy based on molecular dynamics [74], it was also observed that the steady-state creep rate increased with the increase of Cr and Al content, as shown in Figure 7. The creep mechanism also transitions from diffusion creep under low stress to dislocation creep and grain boundary sliding under high stress. However, this simulation is based on the analysis of molecular dynamics at the atomic scale. It can be seen that the stress value is much larger than the experimental value in the experiment, but it still has a strong reference value for revealing the relationship between the creep mechanism and stress.

### 4.2. Stability of ODS FeCrAl Alloys during Creep

**Figure 8:**
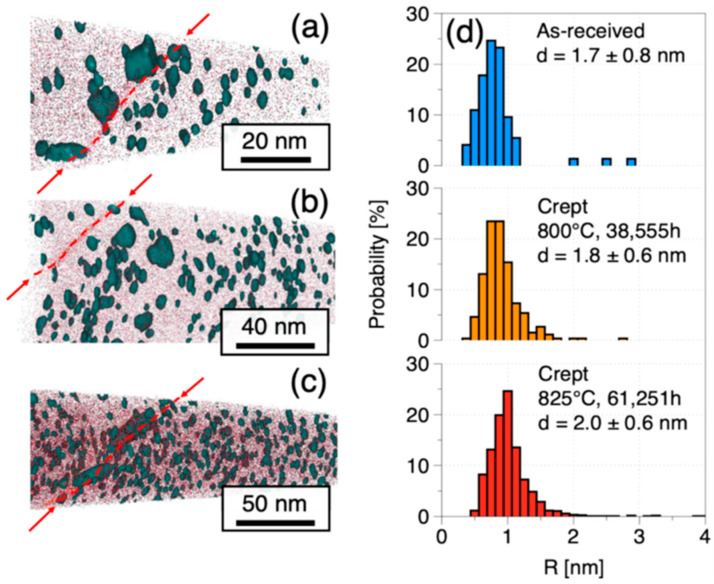
MA957 APT results and dispersed particle size distribution of MA957 sample after long-time creep. (**a**) as received, (**b**) 38,255 h crept, (**c**) 61,251 h crept, grain boundaries are highlighted by red lines and arrows, (**d**) dispersed particle size distribution of different samples.

Although it has been mentioned in previous studies that the grain size of the sample increases under high temperature creep conditions, the effect of grain size on the steady-state creep rate is not significant. However, the change of dispersed particles is more critical to the creep performance. At present, the research on the long-term creep of ODS FeCrAl alloy is relatively lacking, but the stability research of other ODS alloys can also be used for reference. The study of Massey et al. [75] on the long-term creep of MA957 shows that the dispersed particles are quite stable. After creeping for 61,251 h at 825 °C and 70 MPa, the average size, number density and composition of the particles were not affected, as seen in Figure 8. The samples only showed some pores after creep, which may be the main reason for the failure of the samples during subsequent experiments. Oka’s long-term creep experiments on 9Cr and 12Cr ODS alloys also show that the size and structure of the particles are stable, and only 12Cr samples precipitate some carbides at the grain boundaries [76].

Although there is no long-term creep experiment of FeCrAl alloys, the results of a large number of high-temperature corrosion experiments found that the high-temperature stability of ODS FeCrAl alloy is excellent [22,29,77]. Through some high temperature oxidation creep, it can be seen that the FeCrAl alloy is relatively stable throughout the creep process [78,79]. In the existing creep research, no obvious growth phenomenon of oxide-dispersed particles was found.

### 4.3. Creep in Other Coupled Environments

Although thermal creep is a performance evaluation that is more suitable for actual working conditions, in order to further promote the practical application of ODS FeCrAl alloys, it is necessary to test under various service environment coupling conditions. For example, in fast reactor applications, creep in LBE or liquid lead environments must be considered. Studies have shown that FeCrAl alloys can form effective protective films in lead-bismuth eutectic (LBE) at 500–600 °C [80,81]. There are also studies to improve the mechanical properties of samples in liquid LBE by preparing FeCrAl coating on the surface of T91 steel [82]. After studying the corrosion of FeCrAl alloys with different compositions in LBE at 600 °C, Lim [22] proposed the basis for judging whether a protective film can be formed in the alloy, that is, Al wt.% + 0.5Cr wt.% must be greater than 9.8. In addition, FeCrAl alloys with low Cr and high Al performed better than high Cr and low Al in LBE at 500–600 °C because higher Cr content would lead to the precipitation of brittle phases. It can be seen that the stability of the ODS FeCrAl alloy in LBE is very good. However, there are few studies on the creep data of ODS FeCrAl alloy in LBE. The creep performance of other ODS alloys in LBE is similar to that of thermal creep [83,84], so the creep performance of ODS FeCrAl alloy in LBE is still worth examining. Another condition in advanced nuclear energy systems is irradiation. The current research [85,86,87] on the radiation resistance of FeCrAl alloys shows that it has a strong similarity with Al-free FeCr alloys. However, data on creep in irradiated environments is very scarce, and more experiments are still needed to evaluate its performance.

In addition to compatibility, another problem that requires attention is the liquid metal embrittlement of the alloy. This phenomenon generally exists in iron-based steel. Studies have shown that the Fe–10Cr–4Al sample does not embrittle in pure liquid Pb under low oxygen environments, but embrittlement is very obvious in LBE, which shows that Bi is the key to its occurrence of LME [88]. However, although the sample was embrittled and the elongation decreased significantly, the yield strength and tensile strength were not affected. The embrittlement of ODS FeCrAl alloy in LBE is also affected by many factors, the first being temperature. The slow strain rate test experiment shows that embrittlement mostly occurs at lower temperatures, that is, between 140–425 °C [88]. When the temperature is further raised above 500 °C, its ductility recovers [89]. Another important factor is whether the oxide film is formed or not. The mass loss of pre-oxidized APMT in LBE at 550–650 °C is very low [90]. It can be seen that when a continuous oxide film is formed on the surface of the alloy, the embrittlement phenomenon weakens, which is directly related to the oxygen concentration in LBE. This suggests that the elongation rate of the sample in an oxygen-poor environment decreases more than that in an oxygen-enriched environment [91]. Since an oxygen-enriched environment LBE can provide sufficient O for the sample to react to form an oxide film, this suitable dissolved O can also repair the defective part of the sample to a certain extent. Studies have also shown that adding a part of Si to ODS FeCrAl alloy will improve its LME performance.

## 5. Conclusions and Outlook

ODS FeCrAl alloy is a promising candidate material for accident tolerant fuel systems and Gen-IV advanced reactors due to its excellent corrosion resistance and improved high temperature strength compared with traditional FeCrAl alloys. Creep performance is a key service property for engineering applications of ODS FeCrAl alloy, while the related mechanism is still not very well understood. This paper provides a comprehensive review of the current state and prospects of the investigation of creep performance of ODS FeCrAl alloy:

1. ODS FeCrAl alloys exhibit significant improvement in creep resistance compared to traditional FeCrAl alloys and have been further improved through composition optimization. Although its creep resistance has no advantage over ODS FeCr alloy at intermediate temperatures, its advantage in creep resistance is evident when the temperature is raised to 1000 °C. The excellent performance of ODS FeCrAl alloys under ultra-high temperature and low stress conditions also makes them highly competitive as candidate materials for accident-tolerant fuel systems and Gen-IV advanced reactors.
2. The quantification of the creep performance of ODS alloys is challenging, especially in ODS FeCrAl alloys, as the system of dispersed oxide particles is more complex than ODS FeCr steels. Different dispersed particles have different interface relationships, which will be reflected differently in the interaction with dislocations. Moreover, samples with different heat treatment histories currently show significantly different creep performance. Therefore, future research on the creep performance of ODS FeCrAl alloy should focus more on samples approaching their final service state.
3. Testing of creep and other service environment coupling conditions is still in a relatively blank stage, but it is critical for the final engineering application of ODS FeCrAl alloys in advanced nuclear systems. Consequently, further research is needed to comprehensively evaluate the performance of ODS FeCrAl alloys as candidate materials for different coolants and under irradiation.

## Figures and Tables

**Table 1:** Nominal composition of FeCrAl alloys in wt.%.

Alloy	Fe	Cr	Al	Mo	Y_2_O_3_	Zr	Ti
APMT [20]	Bal.	22	5	3	0.5	-	-
MA956 [21]	Bal.	20	4.5	-	0.5	-	0.4
PM2000 [22]	Bal.	20	5.5	-	0.5	-	0.5
C26M [23]	Bal.	12	6	2	<0.06	-	-
FeCrAl-ODS-1 [24]	Bal.	12	6	-	0.5	0.4	0.5
FeCrAl-ODS-2 [25]	Bal.	13	5	2	0.35	0.6	0.5

## Data Availability

Not applicable.
